# ApoE−/− PGC-1α−/− Mice Display Reduced IL-18 Levels and Do Not Develop Enhanced Atherosclerosis

**DOI:** 10.1371/journal.pone.0013539

**Published:** 2010-10-22

**Authors:** Sokrates Stein, Christine Lohmann, Christoph Handschin, Elin Stenfeldt, Jan Borén, Thomas F. Lüscher, Christian M. Matter

**Affiliations:** 1 Cardiovascular Research, Institute of Physiology, and Zurich Center for Integrative Human Physiology (ZIHP), University of Zurich and Cardiology, Cardiovascular Center, University Hospital Zurich, Zurich, Switzerland; 2 Biozentrum, University of Basel, Basel, Switzerland; 3 Sahlgrenska Center for Cardiovascular and Metabolic Research, University of Goteborg, Goteborg, Sweden; University of Sheffield, United Kingdom

## Abstract

**Background:**

Atherosclerosis is a chronic inflammatory disease that evolves from the interaction of activated endothelial cells, macrophages, lymphocytes and modified lipoproteins (LDLs). In the last years many molecules with crucial metabolic functions have been shown to prevent important steps in the progression of atherogenesis, including peroxisome proliferator activated receptors (PPARs) and the class III histone deacetylase (HDAC) SIRT1. The PPARγ coactivator 1 alpha (Ppargc1a or PGC-1α) was identified as an important transcriptional cofactor of PPARγ and is activated by SIRT1. The aim of this study was to analyze total *PGC-1α* deficiency in an atherosclerotic mouse model.

**Methodology/Principal Findings:**

To investigate if total PGC-1α deficiency affects atherosclerosis, we compared *ApoE^−/−^ PGC-1α^−/−^* and *ApoE^−/−^ PGC-1α^+/+^* mice kept on a high cholesterol diet. Despite having more macrophages and a higher ICAM-1 expression in plaques, *ApoE^−/−^ PGC-1α^−/−^* did not display more or larger atherosclerotic plaques than their *ApoE^−/−^ PGC-1α^+/+^* littermates. In line with the previously published phenotype of *PGC-1α^−/−^* mice, *ApoE^−/−^ PGC-1α^−/−^* mice had marked reduced body, liver and epididymal white adipose tissue (WAT) weight. VLDL/LDL-cholesterol and triglyceride contents were also reduced. Aortic expression of *PPARα* and *PPARγ*, two crucial regulators for adipocyte differentitation and glucose and lipid metabolism, as well as the expression of some PPAR target genes was significantly reduced in *ApoE^−/−^ PGC-1α^−/−^* mice. Importantly, the epididymal WAT and aortic expression of *IL-18* and IL-18 plasma levels, a pro-atherosclerotic cytokine, was markedly reduced in *ApoE^−/−^ PGC-1α^−/−^* mice.

**Conclusions/Significance:**

*ApoE^−/−^ PGC-1α^−/−^* mice, similar as *PGC-1α^−/−^* mice exhibit markedly reduced total body and visceral fat weight. Since inflammation of visceral fat is a crucial trigger of atherogenesis, decreased visceral fat in *PGC-1α*-deficient mice may explain why these mice do not develop enhanced atherosclerosis.

## Introduction

Atherosclerosis is a chronic inflammatory disease that results from interaction between activated endothelial cells, modified low-density lipoproteins (LDL), monocyte-derived macrophages, T cells, and the vessel wall. Activated endothelial cells express adhesion molecules that attract and recruit blood monocytes and lymphocytes. Upon binding to the endothelial layer, these monocytes transmigrate into the subintimal space, and differentiate into macrophages. Plaque macrophages interact with lymphatic cells, mainly T cells, ingest modified LDL via scavenger receptors and become foam cells, thereby promoting plaque formation [1].

PGC-1α was the first described member of the small PGC-1 family of coactivators [2]. Other members of this protein family are PGC-1β and PGC-related coactivator (PRC). PGC-1α is an important cofactor in the transcriptional regulation of genes encoding metabolic enzymes and mitochondrial proteins [3], and it is interacting with many different transcription factors, such as peroxisome proliferator activated receptors (PPARs, including PPARα, PPARβ/δ, and PPARγ), Liver X receptor α and β (LXRα and LXRβ), Glucagon receptor (GR), and Forkhead box O1 (FoxO1) [4], [5], [6], [7], [8], [9], [10].

The phenotype of PGC-1α knock-out mice underlines the central role of this transcription cofactor in homeostatic control of metabolism: they are leaner than wild-type (WT) littermates, have markedly reduced body fat content, and are resistant to diet-induced obesity, hence protected from developing insulin resistance and impaired glucose tolerance [11]. This difference is explained by their CNS-linked hyperactivity and is not a consequence of altered food intake [11].

Overexpression of PGC-1α in human aortic smooth muscle and endothelial cells *in vitro* has been shown to prevent reactive oxygen species (ROS) production and NAD(P)H oxidase activity, with subsequently reduced NF-κB activity and lower expression levels of MCP-1 and VCAM-1 [12], which are important triggers of inflammation and atherosclerosis. Moreover, PGC-1α overexpression in endothelial cells prevented alpha-linoleic acid-induced ROS formation *in vitro* and improved endothelial dysfunction in aortic rings *ex vivo* [13].

The following studies suggest a link between PGC-1α and atherogenesis at the clinical level: Xie et al. reported a correlation between PGC-1α polymorphism and hypertension [14], and Zhang et al. showed an association between PGC-1α polymorphism and the prevalence of coronary artery disease [15].

Thus, we investigated the effects of *PGC-1α* deficiency on atherogenesis by comparing *ApoE^−/−^ PGC-1α^−/−^* and *ApoE^−/−^ PGC-1α^+/+^* mice.

## Results

### Total *PGC-1α^−/−^* deletion does not affect atherogenesis

**Figure 1:**
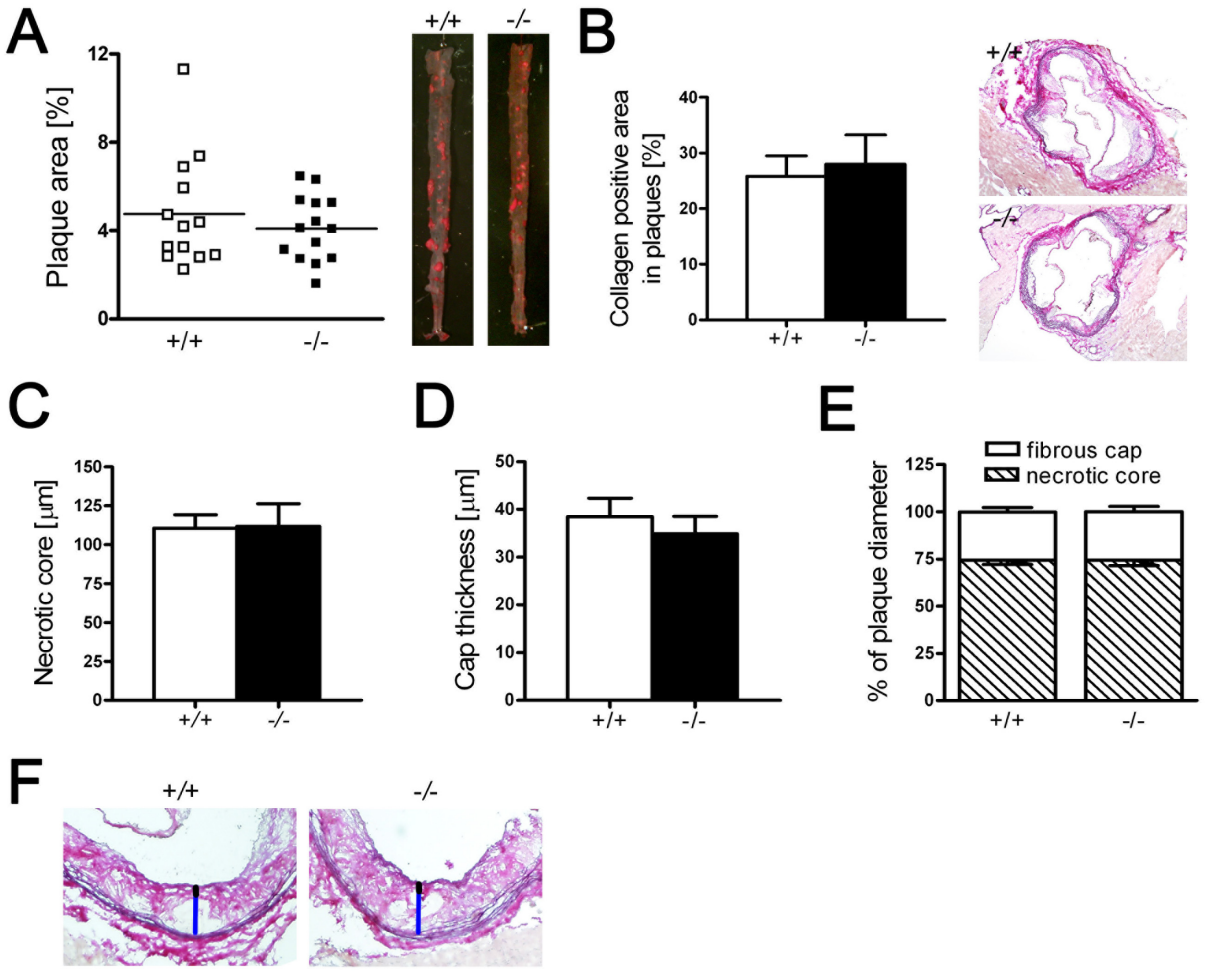
Atherosclerotic lesions and features of plaque vulnerability in *ApoE^−/−^ PGC-1α^−/−^* and *ApoE^−/−^ PGC-1α^+/+^* mice. *En face* plaque quantification of thoraco-abdominal aortae stained with ORO (A). Absolute values of plaque collagen content (B), necrotic core size (C) and cap thickness (D) in plaques from the aortic sinus. Relative values of the necrotic core and fibrous cap size on plaque diameter (E), and representative images to show how the necrotic core (blue line) and fibrous cap (black line) in plaques from the aortic sinus was measured (F). A: *ApoE^−/−^ PGC-1α^+/+^* n = 13 (open circles); *ApoE^−/−^ PGC-1α^−/−^* n = 14 (closed circles). B-E: n = 10. *ApoE^−/−^ PGC-1α^−/−^* (*−/−*) and *ApoE^−/−^ PGC-1α^+/+^* (+/+).

To study the potential role of PGC-1α in atherogenesis, we crossed *PGC-1α^−/−^* with *ApoE^−/−^* mice, and compared 20-week old male *ApoE^−/−^ PGC-1α^−/−^* and *ApoE^−/−^ PGC-1α^+/+^* mice that were kept on a high-cholesterol diet for 12 weeks. Histomorphometry of thoraco-abdominal aortae stained with Oil-Red O (ORO) revealed no difference in atherosclerotic plaque area between *ApoE^−/−^ PGC-1α^−/−^* and *ApoE^−/−^ PGC-1α^+/+^* mice (Fig. 1A). Advanced plaque parameters also revealed a similar total collagen content, plaque diameter or cap thickness in plaques of the aortic sinus that were stained with Elastica van Gieson (Fig. 1B-F).

### Increased macrophage and ICAM-1 expression in *ApoE^−/−^ PGC-1α^−/−^* mice

**Figure 2:**
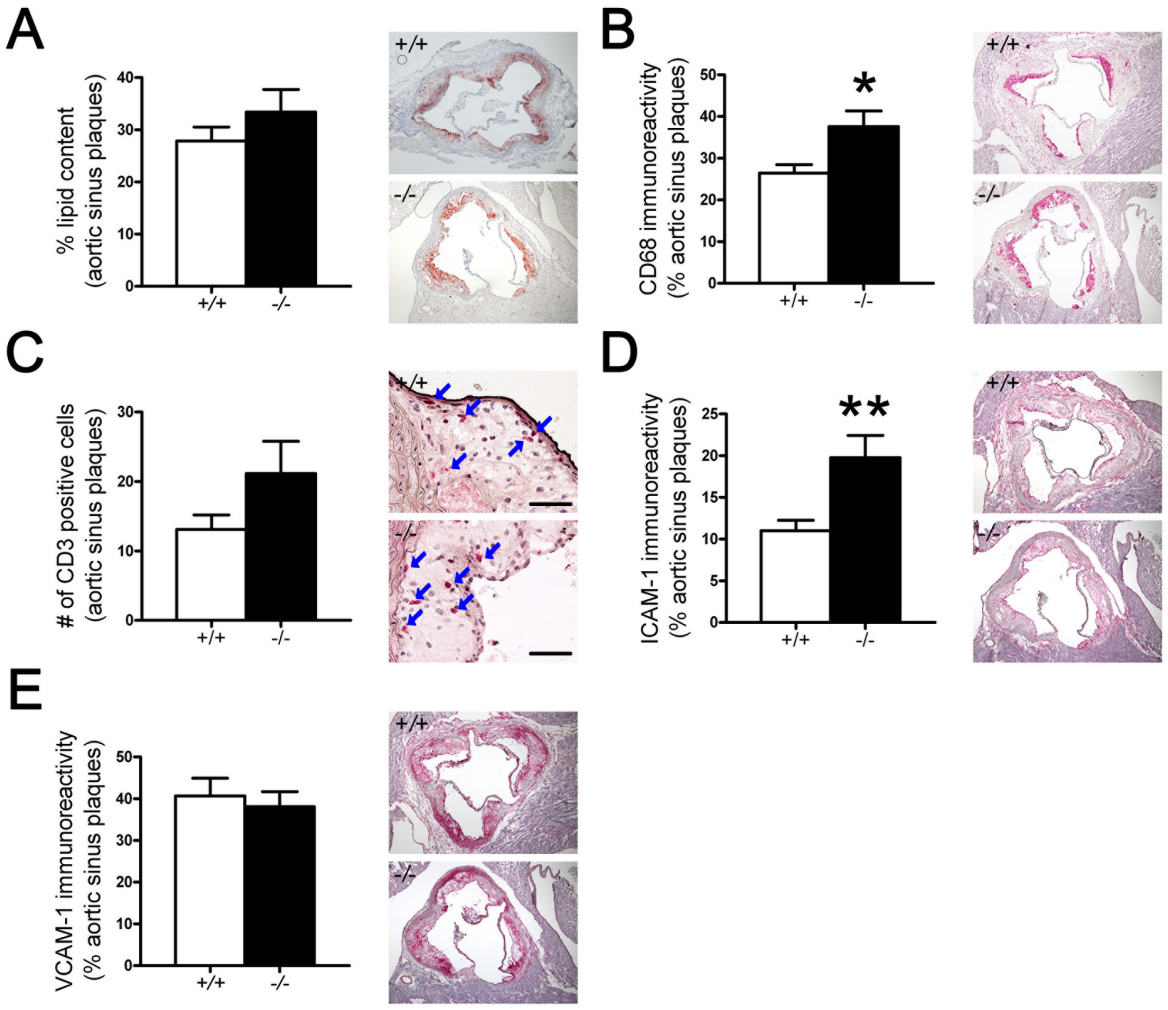
Characterization of plaque inflammation. Quantitative analysis of lipid content in aortic sinus (A; Oil red-O staining), macrophage immunoreactivity (B; CD68-positive cells), T cell number (C; CD3-positive cells; scale bar, 200 µm), VCAM-1 (D) and ICAM-1 (E) immunoreactivity in plaques of the aortic sinus of *ApoE^−/−^ PGC-1α^−/−^* (*−/−*) and *ApoE^−/−^ PGC-1α^+/+^* (+/+) mice expressed as a proportion of the total plaque areas. n = 10 per genotype. * p<0.05. **p<0.01.

To further analyze cellular and molecular mediators in the progression of atherosclerosis, we quantified the amount of lipids, macrophages, T cells, as well as of the adhesion molecules ICAM-1 and VCAM-1 in plaques from the aortic sinus. No difference in lipid content, CD3-positive T cells, and VCAM-1 expression was observed between *ApoE^−/−^ PGC-1α^−/−^* and *ApoE^−/−^ PGC-1α^+/+^* mice. However, more CD68-positive macrophages and ICAM-1-expressing cells were detected in plaques from *ApoE^−/−^ PGC-1α^−/−^* mice (Fig. 2).

### *ApoE^−/−^ PGC-1α^−/−^* mice exhibit reduced total body weight, epididymal white adipose tissue weight, and VLDL/LDL-cholesterol and VLDL/LDL-triglyceride contents

**Figure 3:**
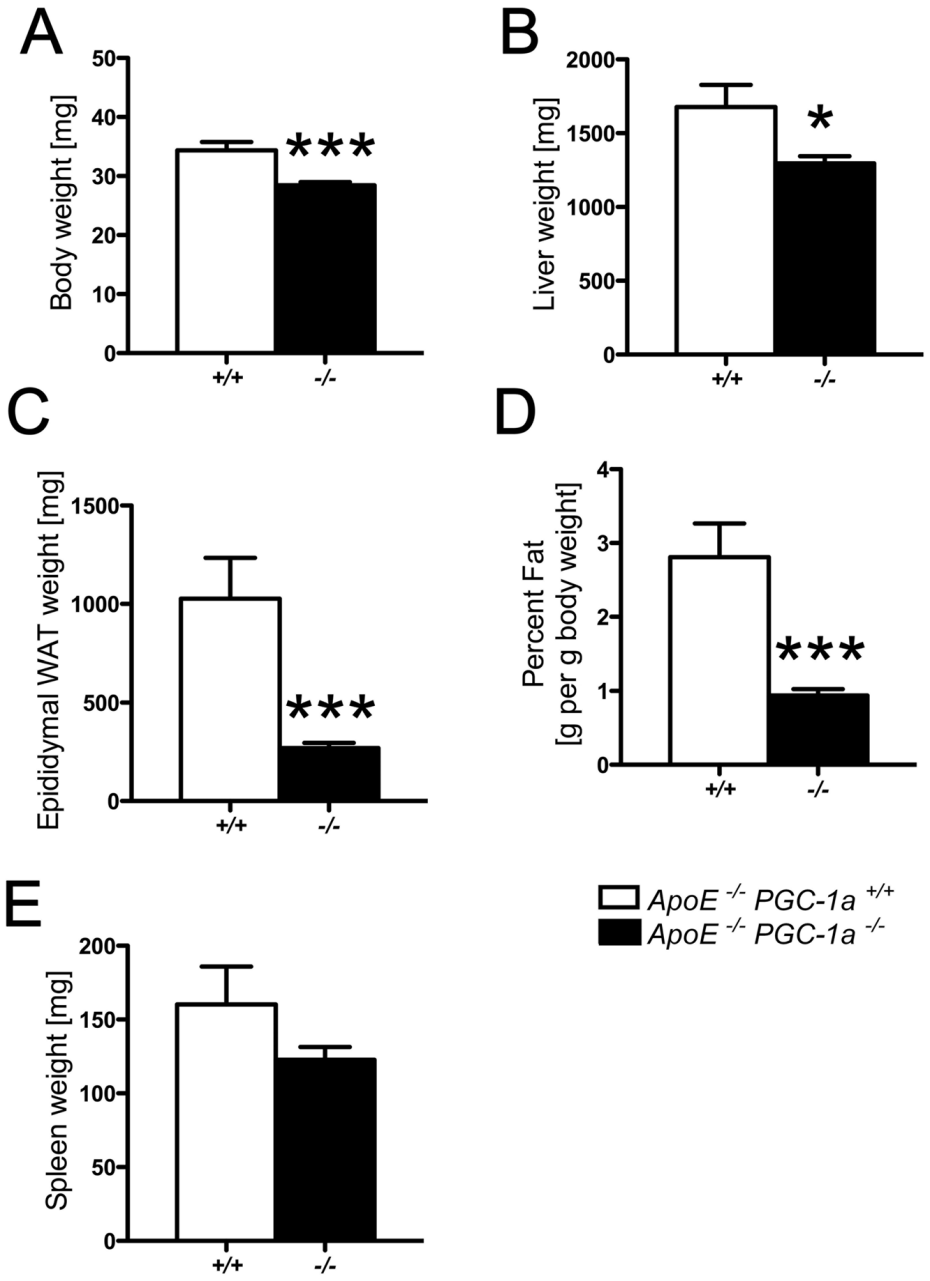
Total body weight and adipose tissue mass. *ApoE^−/−^ PGC-1α^−/−^* exhibit a lower body weight (A), liver weight (B), as well as total epididymal (C) and percent epididymal fat of body weight (D) than *ApoE^−/−^ PGC-1α^+/+^* mice. No difference is observed in spleen weight (E). n≥14 per genotype. * p<0.05; *** p<0.001.

**Figure 4:**
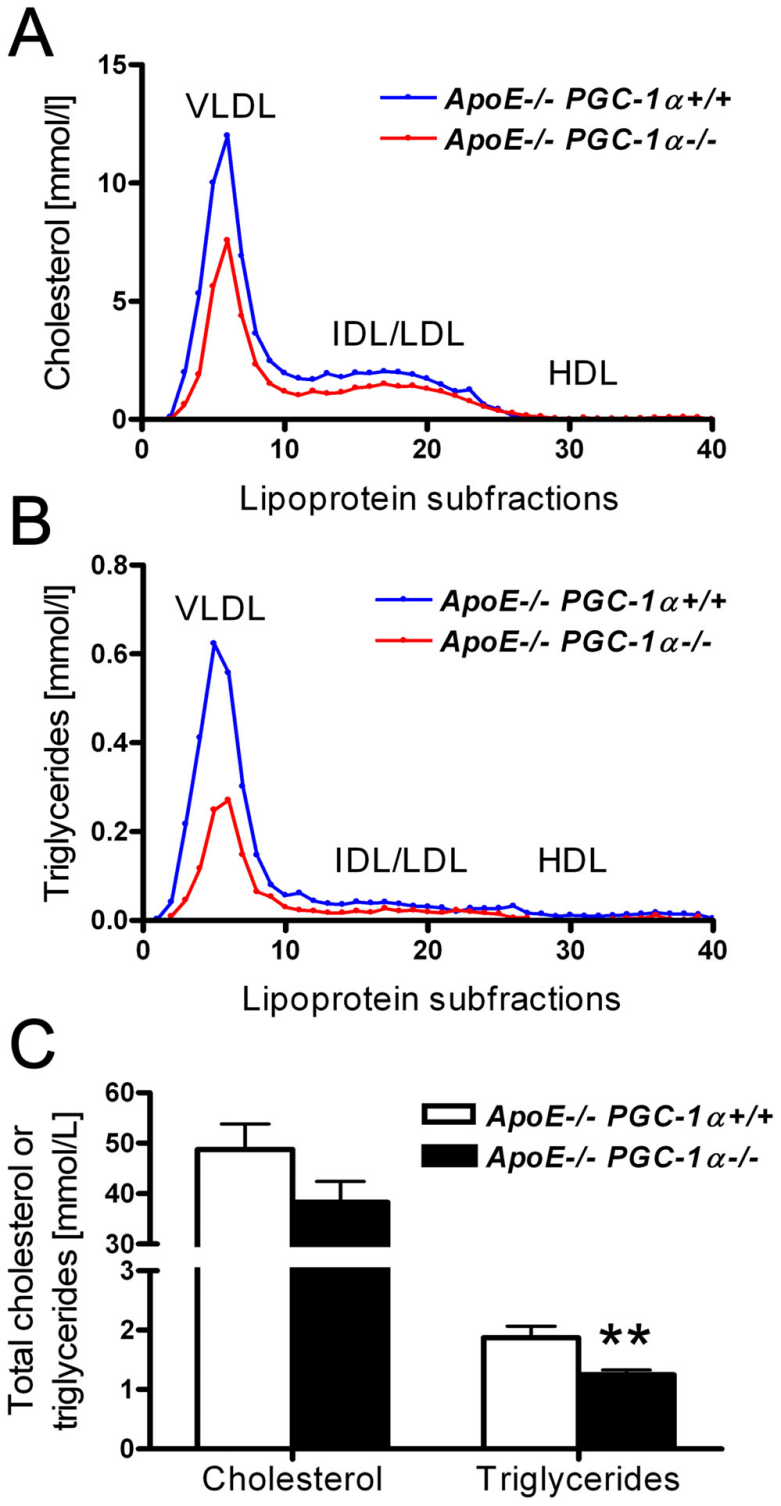
Plasma lipid levels. (A, B) Cholesterol and triglyceride distribution in the plasma lipoprotein fractions of *ApoE^−/−^ PGC-1α^−/−^* and *ApoE^−/−^ PGC-1α^+/+^* mice. Plasma samples were pooled (n = 14 per genotype) and fractionated on a HPLC column. (C) Total cholesterol and triglycerides concentrations were measured with an enzymatic colorimetric assay. n = 14 per genotype. *HPLC*, high pressure liquid chromatography; *HDL*, high-density lipoproteins; *IDL*, intermediate-density lipoproteins; *LDL*, low-density lipoproteins; *VLDL*, very-low-density lipoproteins. ** p<0.01.

*ApoE^−/−^ PGC-1α^−/−^* mice had a lower body, liver, and epididymal fat weight than *ApoE^−/−^ PGC-1α^+/+^* mice (Fig. 3A–D). Spleen weight did not differ between the two groups (Fig. 3E). These data match the published phenotype of *PGC-1α^−/−^* mice [11]. We next analyzed total cholesterol and triglyceride plasma levels and their distribution in lipoprotein fractions. Both cholesterol and triglyceride contents were lower in VLDL and IDL/LDL particles, whereas their content in HDL particles did not differ (Fig. 4A, B). Total plasma cholesterol showed a clear trend, whereas total triglyceride levels were markedly lower in *ApoE^−/−^ PGC-1α^−/−^* compared to *ApoE^−/−^ PGC-1α^+/+^* mice (Fig. 4C).

### Reduced expression of *PPAR* and PPAR target genes

**Figure 5:**
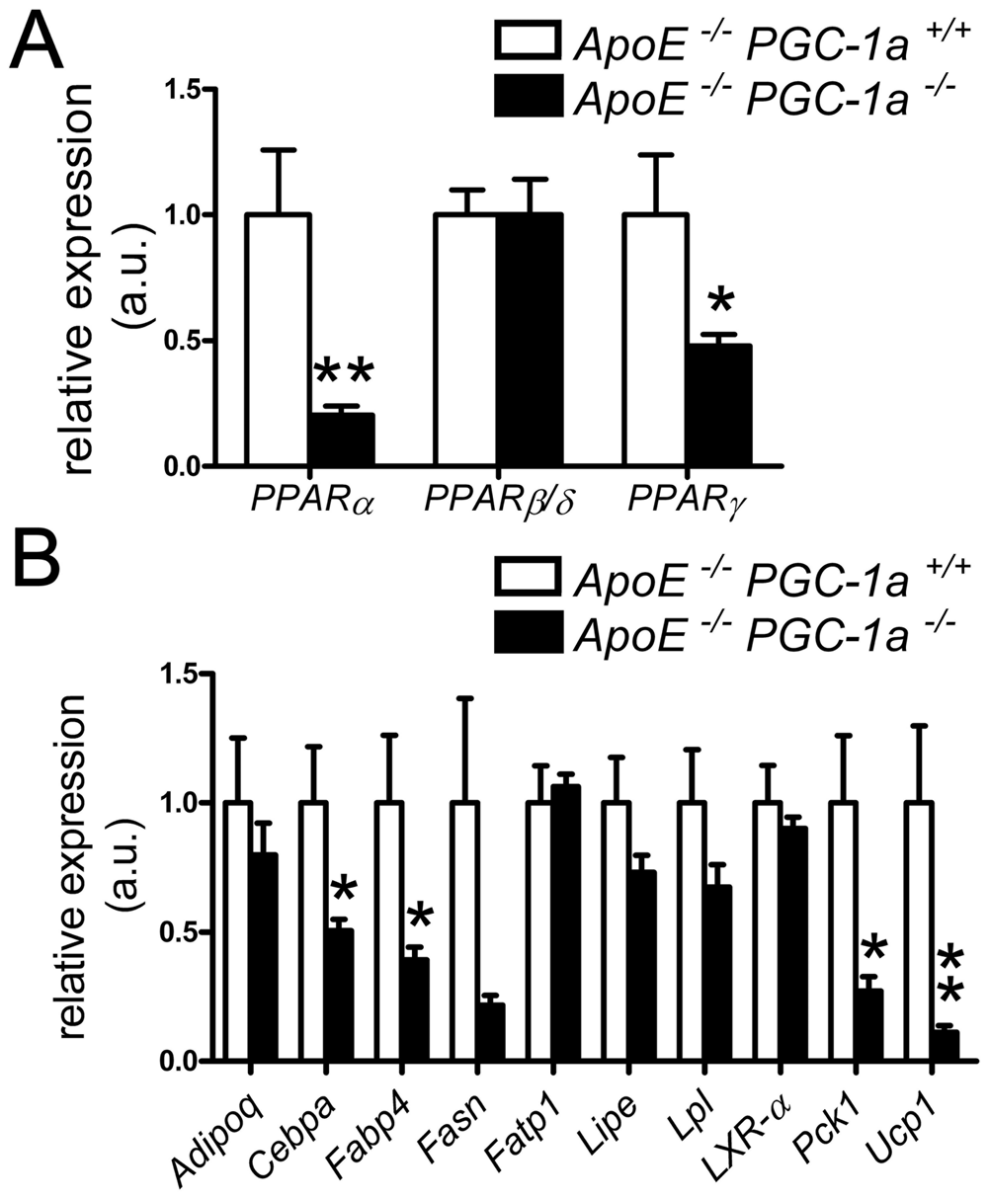
Expression of PPAR and PPAR target genes. (A) Reduced aortic mRNA expression of *PPARα* and *PPARγ*, but unchanged expression of *PPARβ/δ* in *ApoE^−/−^ PGC-1α^−/−^* compared to *ApoE^−/−^ PGC-1α^+/+^* mice. (B) Aortic mRNA expression of *Adipoq, Cebpa, Fabp4, Fasn, Fatp, Lipe, Lpl, LXR-α, Pck1*, and *Ucp1*. n≥9 per genotype. * p<0.05; ** p<0.01.

Peroxisome proliferator activated receptors (PPARs) are important regulators of adipocyte differentiation as well as lipid metabolism and inflammation and their transcription is regulated by PGC-1α [6], [16], [17]. mRNA expression *PPARα* and *PPARγ* was reduced in aortic lysates of *ApoE^−/−^ PGC-1α^−/−^* mice (Fig. 5A), whereas PPARβ/δ levels were not changed (Fig. 5A). To examine if the differential expression of these transcriptional regulators exert functional effects, we quantified the expression of some PPARα and/or PPARγ target genes: *Adipoq (adiponectin), Cebpa (C/EBP-α), Fabp4 (aP2), Fasn (Fatty acid synthase), Fatp1 (Fatty acid transport protein 1), Lipe (Hormone-sensitive lipase), Lpl (Lipoprotein lipase), LXR-α (Liver X receptor α), Pck1 (Phosphoenolpyruvate carboxykinase 1)*, and *Ucp1 (Uncoupling protein 1)*. Expression of *Cebpa, Fabp4, Pck1*, and *Ucp1* was significantly lower in *ApoE^−/−^ PGC-1α^−/−^* compared to *ApoE^−/−^ PGC-1α^+/+^* mice, while the expression of *Fasn* showed the same trend and mRNA levels of *Adipoq, Fatp1, Lipe, Lpl*, and *LXR-α* did not differ (Fig. 5B). These data suggest that *PPARα* and *PPARγ* expression and function may at least in part be suppressed in *ApoE^−/−^ PGC-1α^−/−^* mice.

### Expression of *IL-18* in epididymal WAT from *ApoE^−/−^ PGC-1α^−/−^* mice is markedly reduced

**Figure 6:**
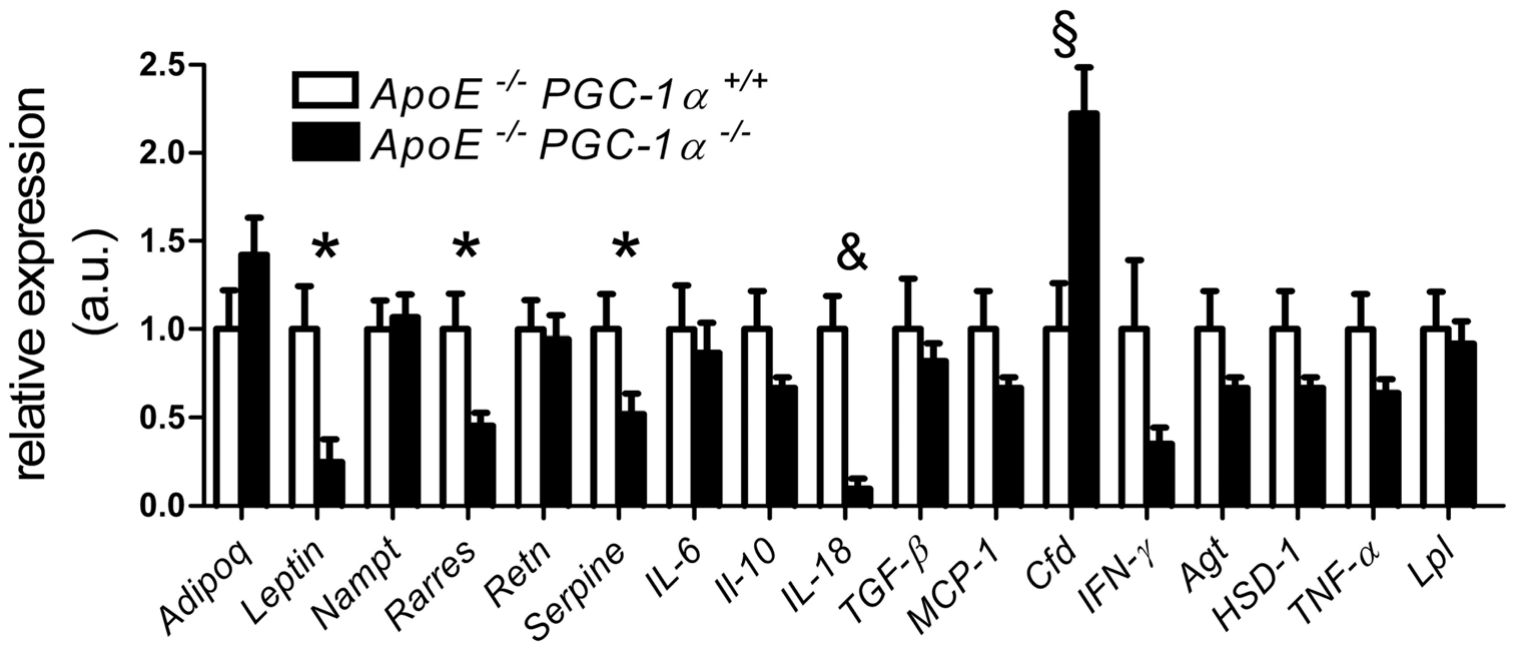
Expression of adipocyte-derived hormones and adipokines. Epididymal WAT mRNA expression in ApoE*^−/−^* PGC-1α*^−/−^* and ApoE*^−/−^* PGC-1α^+/+^ mice: Adipoq, Leptin, Nampt, Rarres2, Retn, Serpine1, IL-6, IL-10, IL-18, TGFβ, MCP-1, Cfd, IFN-γ, Agt, 11β-HSD1, TNFα and Lpl. n = 11 per genotype. * p<0.05; § p<0.01, & p<0.001.

Mice transplanted with visceral fat develop more atherosclerosis than sham-operated animals [18], supporting the clinical concept that that visceral fat as well as its inflammatory mediators are an important risk factors of atherosclerosis and acute coronary events [19], [20]. We therefore analyzed the expression of adipose tissue-derived hormones and cytokines in *ApoE^−/−^ PGC-1α^−/−^* and *ApoE^−/−^ PGC-1α^+/+^* mice. While expression of *Adipoq, Nampt (Nicotinamide phosphoribosyltransferase), Retn (Resistin), IL-6, IL-10, TGF-β, MCP-1, IFN-γ, Agt (Angiotensinogen), 11β-HSD1 (11-beta-hydroxysteroid dehydrogenase 1), TNFα, and Lpl* was only mildly reduced or unchanged, the expression of *leptin, Rarres2 (chemerin), Serpine1 (PAI-1), and IL-18* was lower, and expression of *complement factor D (Cfd* or *adipsin)* higher in *ApoE^−/−^ PGC-1α^−/−^* compared to *ApoE^−/−^ PGC-1α^+/+^* epididymal WAT (Fig. 6).

### Reduced expression of IL-18 and CXL16 in aortic lysates from *ApoE^−/−^ PGC-1α^−/−^* mice

**Figure 7:**
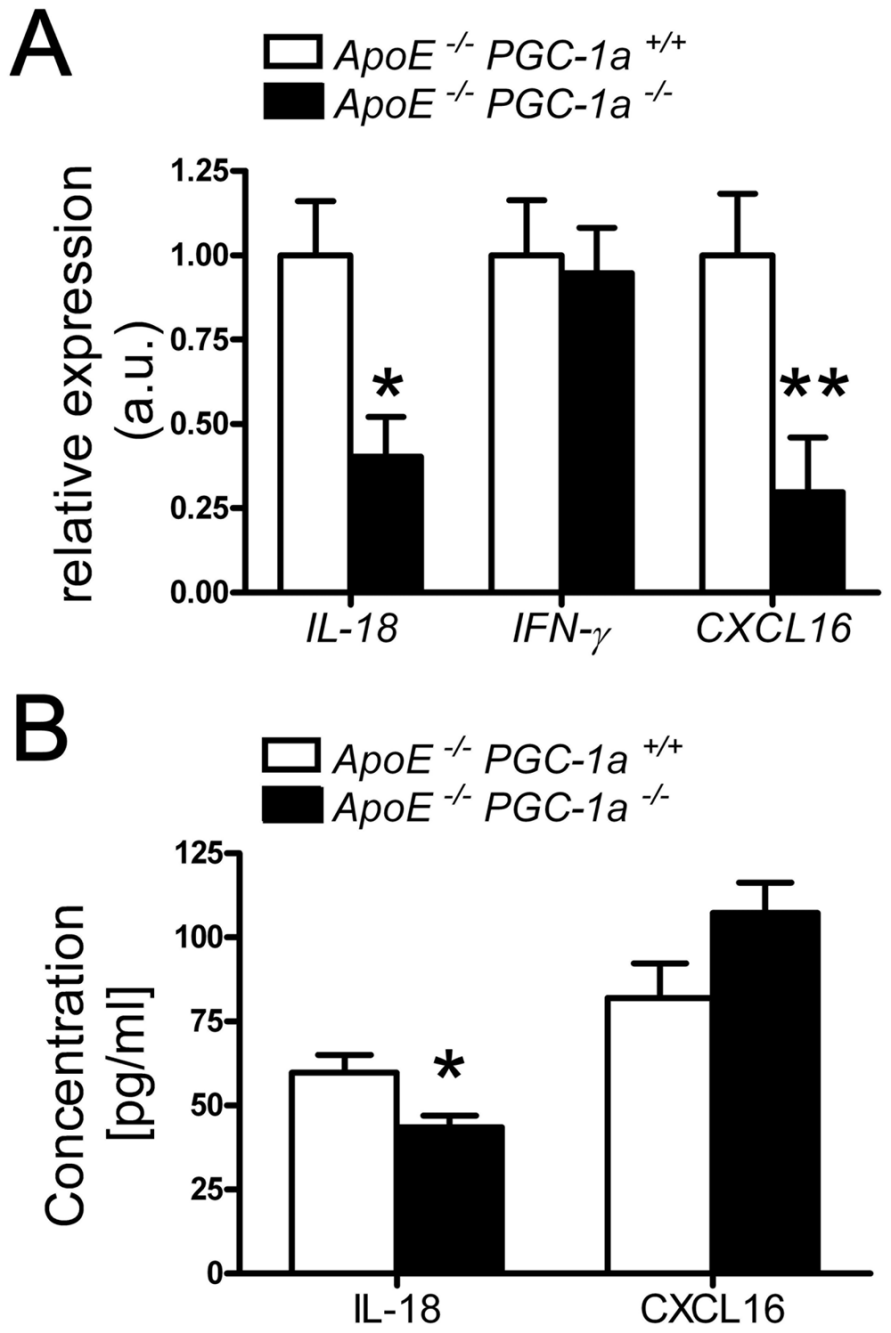
Aortic and plasma expression levels of *IL-18* and *CXCL16*. (A) Reduced aortic mRNA expression of *IL-18* and *CXCL16*, but no change in the expression of *IFN-γ* is observed in *ApoE^−/−^ PGC-1α^−/−^* compared to *ApoE^−/−^ PGC-1α^+/+^* mice. n≥9 per genotype. (B) In plasma samples only IL-18, but not CXCL16 protein levels differed between *ApoE^−/−^ PGC-1α^−/−^* and *ApoE^−/−^ PGC-1α^+/+^* mice. n≥10 per genotype. * p<0.05; ** p<0.01.

The reduced expression of *IL-18* in epididymal WAT is of special interest, since *ApoE^−/−^ IL-18^−/−^* mice develop less atherosclerosis than control *ApoE^−/−^* mice [21]. Importantly, injection of IL-18 into *SCID/apoE* kockout mice elevated levels of IFN-γ and scavenger receptor for phosphatidylserine and oxidized lipoprotein/CXC chemokine ligand 16 (SR-PSOX/CXCL16) in atherosclerotic lesions [22]. Measurement of these factors in aortic tissue, revealed that *IL-18* and SR-PSOX/CXCL16 mRNA levels were reduced in *ApoE^−/−^ PGC-1α^−/−^* mice, while *IFN-γ* expression did not differ between the two genotypes (Fig. 7A). We also quantified the amount of IL-18 and soluble SR-PSOX/CXCL16 in plasma samples. In line with the reduced expression in epididymal WAT and aortae, IL-18 protein level was also reduced in the plasma of *ApoE^−/−^ PGC-1α^−/−^* compared to *ApoE^−/−^ PGC-1α^+/+^* mice (Fig. 7B). In contrast, plasma levels of secreted SR-PSOX/CXCL16 did not differ between the two genotypes (Fig. 7B).

## Discussion

Our data show that *ApoE^−/−^ PGC-1α^−/−^* and *ApoE^−/−^ PGC-1α^+/+^* mice do not differ with regard to atherosclerosis, features of plaque vulnerability, expression of VCAM-1, and T cells number. Increased expression of ICAM-1 or CD68-positive cells in plaques of *ApoE^−/−^ PGC-1α^−/−^* do not appear to play a substantial role as they do not affect plaque size. Importantly, the double knockout mice are leaner, have lighter liver and epididymal fat, and less cholesterol and triglycerides in VLDL and LDL subfractions. In addition, aortic expression of *PPARα* and *PPARγ* as well as some of their target genes is reduced in *ApoE^−/−^ PGC-1α^−/−^* mice.

This phenotype is in line with the first study that described the phenotype of *PGC-1α^−/−^* mice, which also have markedly reduced body fat content [11]. Because visceral (epididymal) WAT inflammation contributes to disease progression [18], it is not astonishing that we observed no difference in plaque lesions between *ApoE^−/−^ PGC-1α^−/−^* and *ApoE^−/−^ PGC-1α^+/+^* mice. Beyond this notion, our data propose that total *PGC-1α* deficiency may rescue an increased atherosclerotic phenotype because of the reduced paracrine effects mediated by the visceral fat.

The lower aortic expression of *PPARα* and *PPARγ* as well as of PPAR target genes proposes that the function of these two PPARs is suppressed in *ApoE^−/−^ PGC-1α^−/−^* mice. Interestingly, both PPARα and PPARγ can exert anti-atherogenic functions in the arterial wall. For example, administration of the PPARγ ligand rosiglitazone reduces foam cell formation and atherosclerosis in *LDL-R* knockout mice [23], and transplantation of PPARγ-deficient bone marrow into recipient *LDL-R* knockout mice enhanced atherosclerosis [24]. One of the main atherogenic targets of PPARγ is *LXRα* [24], [25], whose expression was not changed between *ApoE^−/−^ PGC-1α^−/−^* and *ApoE^−/−^ PGC-1α^+/+^* aortic lysates.

Reduced expression of *Rarres2 (chemerin), Serpine1 (PAI-1), and IL-18* in visceral adipose tissue could be sufficient to avoid increased atherogenesis. Rarres2 is associated with white adipose tissue inflammation and promotes mobilization and chemotaxis of dendritic cells and macrophages [26], [27]. While its expression correlates with inflammatory markers, such as C-reactive protein, it does not predict atherosclerosis in humans [28]. Nevertheless, an atherogenic contribution of Rarres2 cannot be excluded.

PAI-1 is an anti-fibrinolytic enzyme and has beneficial and deleterious effects in atherogenesis. For example, *PAI-1-*deficient mice showed attenuated neointima formation after perivascular cuff-induced injury [29], and local *PAI-1* overexpression prevented the development of abdominal aortic aneurysm [30]. On the other hand, PAI-1 levels are elevated in various cardiovascular diseases and associated with atherothrombosis [31].

The lowest expression of the tested cytokines in the visceral WAT of *ApoE^−/−^ PGC-1α^−/−^* mice was observed for IL-18. IL-18 is a pro-atherogenic cytokine: Overexpression of IL-18 binding protein and direct injection of recombinant IL-18 accelerate atherogenesis, whereas IL-18 deficiency diminishes plaque formation in *ApoE^−/−^* mice [21], [22], [32], [33]. Furthermore, elevated levels of plasma IL-18 are observed in patients with previous myocardial infarction and are associated with the extent of coronary atherosclerosis [34], [35]. We did not only observe a reduced expression of IL-18 in epididymal WAT, but also in aortic tissue and plasma samples of *ApoE^−/−^ PGC-1α^−/−^* mice. It is conceivable that the lower expression of IL-18 alone is sufficient to avoid an acceleration of atherogenesis in our *ApoE^−/−^ PGC-1α^−/−^* mouse model.

Interestingly, IL-18-mediated increase of atherosclerosis is accompanied by elevation of SR-PSOX/CXCL16 expression [22]. SR-PSOX/CXCL16 is a membrane-bound scavenger receptor that binds to the chemokine (C-X-C motif) receptor 6 on lymphocytes [36], [37], [38], [39]. This membrane-anchored chemokine can be further cleaved by specific proteases, hence released in a soluble form [40], [41], which has been proposed as a biomarker for acute coronary syndromes [42]. We observed reduced mRNA levels of SR-PSOX/CXCL16 in epididymal WAT and aortae of *ApoE^−/−^ PGC-1α^−/−^* mice. However, protein levels of the soluble form of SR-PSOX/CXCL16 in plasma did not differ between *ApoE^−/−^ PGC-1α^−/−^* and *ApoE^−/−^ PGC-1α^+/+^* mice, suggesting that the proteolytic cleavage of this chemokine is not affected in *ApoE^−/−^ PGC-1α^−/−^* mice.

*Cfd* encodes adipsin, the mouse homolog of human complement factor D, which is a serine protease that cleaves factor B in the alternative complement pathway, and it is secreted at high levels in adipose tissue [43], [44], [45]. While adipsin expression is increased in catabolic conditions such as fasting, it is down-regulated in different models of genetic and acquired obesity [46]. In line with these observations, epididymal WAT expression of *adipsin* was higher in *ApoE^−/−^ PGC-1α^−/−^* compared to *ApoE^−/−^ PGC-1α^+/+^* mice. Expression of adipsin and other components of the alternative complement pathway correlate with atherosclerosis [47], suggesting that the elevation of adipsin in *ApoE^−/−^ PGC-1α^−/−^* provides a pro-atherogenic contribution.

Atherosclerosis is a disease combining the complexity of lipid/lipoprotein and inflammatory/immune disorders [48]. Since PGC-1α is affecting these two important atherogenic systems, it is difficult to dissect the functions of this enzyme in the chosen animal model. For example, the reduced body weight and VLDL/LDL-cholesterol and triglyceride contents as well as the diminished expression of IL-18 are certainly anti-atherogenic, whereas the increased expression of adipsin may play a pro-atherogenic role in *ApoE^−/−^ PGC-1α^−/−^* mice. Further studies using tissue-specific *PGC-1α* knockout or overexpression will be necessary to address these questions in more detail.

## Materials and Methods

### Animals

*ApoE^−/−^* C57BL/6 [49] mice were crossed to *PGC-1α^−/−^* C57BL/6 [11], to generate *ApoE^−/−^ PGC-1α^−/−^* mice and *ApoE^−/−^ PGC-1α^+/+^* littermates. Of those, male mice were fed a high-cholesterol diet (D12108: 40 kcal% fat, 1.25% cholesterol, Research Diets Inc.) for 12 weeks starting at the age of 8 weeks. Mice were weighted before being sacrificed, and biopsies of aortae, heart, liver, spleen, brown and white adipose tissue, and pancreas frozen in liquid nitrogen or OCT (Optimal Cutting Temperature) for later analyses.

### Ethics Statement

All animal procedures were approved by the local animal committee (Kantonales Veterinäramt Zürich, protocol no. 171/2006) and performed in accordance with our institutional guidelines.

### Immunohistochemistry

5 mm serial cryosections from the aortic sinus were stained with rat anti-CD68, rat anti-CD3 (Abcam), rat anti-VCAM-1 (BD Biosciences), rat anti-ICAM-1 (Serotec), or oil-red O (ORO). Thoraco-abdominal aortae were fixed with 4% paraformaldehyde and plaques stained with ORO for *en face* analysis. Collagen, fibrous cap thickness, and necrotic core size were analyzed on Elastica van Gieson (EVG)-stained cryosections of the aortic sinus as described [50], [51]. Means were taken from n = 10 different mice evaluating 6 serial cryosections/tissue from each mouse.

### RNA and protein analysis

Total RNA isolated from proximal aortae was extracted with TRIZOL (Invitrogen), reverse transcribed with Ready-To-Go You-Prime First-Strand Beads (GE Healthcare), and the cDNA (n≥9 per genotype) quantified by qPCR using SYBR Green JumpStart Taq ReadyMix (Sigma-Aldrich). Primer sequences can be found in the supplemental Table S1.

### IL-18 and CXCL16 ELISA

Quantification of IL-18 and CXCL16 in plasma of mice where performed with Mouse IL-18 Platinum ELISA kit (Bender MedSystems) and Mouse CXCL16 ELISA kit (RayBiotech) according to the manufacturers instructions. Plasma was diluted 1∶2 for the IL-18, and 1∶32 for the CXCL16 ELISA assay.

### Cholesterol, triglycerides, and lipoprotein subfractioning

Total plasma cholesterol and triglycerides were quantified using Infinity Cholesterol TR13421 and Infinity Triglycerides TR22421 (Thermo Electron Cooperation), respectively. The lipid distribution in plasma lipoprotein fractions was assessed by fast-performance liquid chromatography gel filtration with a Tricorn Superose 6 10/300 GL column (GE Healthcare) [52].

### Statistical analyses

Data are presented as mean ± SEM. The *en face* ORO quantification was analyzed using a non-parametric Mann-Whitney U *t*-test. Statistical significance of differences of all other experiments was calculated using an unpaired Student's *t*-test. Significance was accepted at the level of p<0.05.

## Supporting Information

Table S1 Primer sequences.
(0.08 MB PDF)
