# Supplementary material for: ApoE−/− PGC-1α−/− Mice Display Reduced IL-18 Levels and Do Not Develop Enhanced Atherosclerosis
Source: PLoS One. 2010 Oct 22;5(10):e13539. doi: 10.1371/journal.pone.0013539 (PMC2962638; doi:10.1371/journal.pone.0013539)
Supplement: Table S1 — Primer sequences. (0.08 MB PDF) [file pone.0013539.s001.pdf]

Table S1 - Primer sequences

Mouse gene name	anti/sense	Sequence (5' to 3')
11β-HSD1	S	CACTTATCTGAAGCCTCAAGGGCG
11β-HSD1	A	CCAATCCCTTTGCTGGCCCC
Adiponectin (Adipoq)	S	AATCCTGCCCAGTCATGCCG
Adiponectin (Adipoq)	A	CTTTCCTGCCAGGGGTTCCG
Angiotensinogen (Agt)	S	TGCCCCAGCTGGAAATCCGA
Angiotensinogen (Agt)	A	ATGGCGAACAGGAAGGGGCT
Cebpa (C/EBP-α)	S	GTCACTGGTCAACTCCAGCA
Cebpa (C/EBP-α)	A	TGGACAAGAACAGCAACGAG
Cfd (Adipsin)	S	GCCCTACATGGCTTCCGTGC
Cfd (Adipsin)	A	TGGGGACCCAACGAGGCATT
Cxcl16	S	GGGACAGAAGGCGCCACCAC
Cxcl16	A	CCCTGGTTGCCATCGCCTGG
IFN-γ	S	ATCTGGAGGAACTGGCAAAA
IFN-γ	A	TGAGCTCATTGAATGCTTGG
IL-10	S	CCAAGCCTTATCGGAAATGA
IL-10	A	TTTTCACAGGGGAGAAATCG
IL-18	S	ACAACTTTGGCCGACTTCAC
IL-18	A	TGGATCCATTTCCTCAAAGG
IL-6	S	CCTCTCTGCAAGAGACTTCCATCCA
IL-6	A	AGCCTCCGACTTGTGAAGTGGT
Lep (leptin)	S	CAGCAGCTGCAAGGTGCAAG
Lep (leptin)	A	GCCAGTGACCCTCTGCTTGG
Lipe (Hormone-sensitive lipase)	S	GGGCCTGGCAGTGGTGTGTAAC
Lipe (Hormone-sensitive lipase)	A	TGAGAACGCTGAGGCTTTGATCTTG
Lpl	S	TTTGGCTCCAGAGTTTGACCGC
Lpl	A	CGAAGGTCTTGCTGCTGTGGTT
MCP-1	S	ACTGAAGCCAGCTCTCTCTTCCTC
MCP-1	A	TTCCTTCTTGGGGTCAGCACAGAC
Nampt (visfatin)	S	TCCCGAGGGCTCTGTCATCC
Nampt (visfatin)	A	GCCCCTATGCCAGCAGTCTCT
PAI-1 (Serpine 1)	S	CAGAGGGCCCCTGGAGAAGT
PAI-1 (Serpine 1)	A	ATTGTCTCTGTCGGGTTGTGCC
Pck1 (Pepck)	S	CCAACGTGGCCGAGACTAGCG
Pck1 (Pepck)	A	GGCACATGGTTCCGCGTCCT
PPARα	S	ATGCCAGTACTGCCGTTTTC
PPARα	A	GGCCTTGACCTTGTTCATGT
PPARβ/δ	S	TGGAGCTCGATGACATGTAC
PPARβ/δ	A	GTACTGGCTGTCAGGGTGGT
PPARγ	S	TCCGAAGAACCATCCGATTGAA
PPARγ	A	CATACAAATGCTTTGCCAGGGC
Rarres2 (chemerin)	S	ACCTGTGCAGTTGGCCTTCC
Rarres2 (chemerin)	A	GAGGATCCTGAGGCCCTTGCT
Retn (resistin)	S	CAGCATGCCACTGTGTCCCA
Retn (resistin)	A	CACGAATGTCCCACGAGCCA
TGF-β	S	GGACCGCAACAACGCCATCT
TGF-β	A	CCTTGGTTCAGCCACTGCCG
TNF-α	S	CCCTCACACTCAGATCATCTTCT
TNF-α	A	GCTACGACGTGGGCTACAG
Ucp1 (Uncoupling protein 1)	S	TCAGCTGTTCAAAGCACACA
Ucp1 (Uncoupling protein 1)	A	GTACCAAGCTGTGCGATGTC
